# The association between QT interval changes and the treatment protocols of COVID-19 patients

**DOI:** 10.14744/nci.2022.86836

**Published:** 2022-07-07

**Authors:** Yasar Sertbas, Kamil Ozdil, Sait Terzi, Selma Dagci, Bengu Saylan, Volkan Kizilay, Goktug Savas, Aysun Erdem Yaman, Meltem Sertbas, Habip Yilmaz, Cevdet Ugur Kocogullari

**Affiliations:** 1 Department of Internal Medicine, Health Sciences University, Fatih Sultan Mehmet Training and Research Hospital, Istanbul, Turkiye; 2 Department of Gastroenterology, Health Sciences University, Umraniye Training and Research Hospital, Istanbul, Turkiye; 3 Department of Cardiology, Health Sciences University, Siyami Ersek Training and Research Hospital, Istanbul, Turkiye; 4 Department of Chest Diseases, Health Sciences University, Sultan II. Abdulhamid Han Training and Research Hospital, Istanbul, Turkiye; 5 Department of Statistics, Health Sciences University, Umraniye Training and Research Hospital, Istanbul, Turkiye; 6 Departmant of Anesthesiology and Reanimation, Health Sciences University, Siyami Ersek Training and Research Hospital, Istanbul, Turkiye; 7 Departmant of Cardiovasculer Surgery, Health Sciences University, Siyami Ersek Training and Research Hospital, Istanbul, Turkiye

**Keywords:** COVID-19, favipiravir, hydroxychloroquine, moxifloxacin

## Abstract

**OBJECTIVE:**

This study aimed to investigate the QT, QTc, and QTc dispersion changes that may occur with the use of hydroxychloroquine (HCQ), favipiravir, and moxifloxacin in combination or alone in COVID 19 patients.

**METHODS:**

This study was retrospectively conducted on 193 inpatients diagnosed with COVID-19. We divided the patients into four separate groups due to their medications as, group-1: favipiravir, group-2: favipiravir + HCQ, group-3: favipiravir + moxifloxacin, and group-4: favipiravir + moxifloxacin + HCQ. We recorded their pre and post-treatment QT parameters of each group and evaluated the changes of these parameters with the SPSS statistical program.

**RESULTS:**

The mean age of the patients was 63.1±17.7. In group 1 and 2, although there were slight changes in QT parameters, these results were not statistically significant. In group 3, significant increases in QT and QTc dispersion occurred (p=0.005 and p=0.018). In the 4^th^ group where the triple therapy was applied, there was a significant increase only in the QTc values (p=0.027). When we compared the changes of QT parameters for each group, a significant difference was found in ΔQTc dispersion, and post hoc analysis showed that it was due to changes in the third group (p=0.047).

**CONCLUSION:**

We thought that, if there is a COVID-19 infection with an additional bacterial infection, and if there is a need of using moxifloxacin alone or together with HCQ, additional risk factors that may cause QT interval prolongation should be reviewed and ECG monitoring of the patients should be performed during the treatment period.

## Highlight key points

- COVID-19 pneumonia remains the leading cause of death due to its effects on respiratory and cardiac systems of the patient.
- After the use of moxifloxacin and favipiravir together, significant prolongation of the QT interval may occurred.
- If there is a need of using moxifloxacin alone or together with HCQ, additional risk factors that may cause QT interval prolongation should be reviewed and ECG monitoring of the patients must be performed.

An outbreak of pneumonia that is considered to have developed due to coronavirus disease 19 (COVID-19) caused by the SARS-CoV-2 virus was first reported from Wuhan Hubei Province, Republic of China, in December 2019. The World Health Organization reported COVID-19 as a pandemic disease in March 2020, as the spread of the disease could not be brought under control with a great morbidity and mortality whole around the world. Since then, COVID-19 has spread rapidly with 173.609.772 confirmed cases and 3.742.563 deaths have been seen across the world until June 9, 2021 [1, 2]. COVID-19 pneumonia remains the leading cause of death due to its effects on respiratory and cardiac systems of the patient.

To deal with this emergency situation, several treatment options have been implemented globally based on *in vitro* studies or small observations. Various agents found to be effective in antiviral treatment of COVID-19 as remdesevir, lopinavir/ritonavir, favipiravir, hydroxychloroquine (HCQ), azithromycine, intravenous immunoglobulins, glucocorticoids, and interleukin-6 antagonists [3]. In addition to these agents, if a bacterial co-infection with lung involvement is considered, fluoroquinolone group antibiotics are frequently preferred for adding to the treatment [4]. Apart from the pulmonary and cardiac damages caused by the disease itself, important side effects may also occur due to medications used as treatment. In addition to temporary elevations in liver or kidney function tests, these medications may also cause myocardial damage and life-threatening cardiac arrhythmias.

The QT interval corresponds to the total duration of ventricular depolarization and repolarization. The variation in duration of the QT interval on the surface ECG is referred to as QT dispersion (QTd) [4]. Extended QTc interval and QTc dispersion are potential risk factors for malignant ventricular arrhythmias affecting mortality in various patient groups. One major concern of hydrochloroquine and moxifloxacin is the possibility of cardiac side effects such as QTc prolongation and torsades de pointes (Tdp). In the meantime, there are also some studies on the issue that favipiravir might also cause QT prolongation [5].

In our study, we aimed to investigate the ECG changes including QT, QTc, QTc dispersion, that may occur with the use of HCQ, favipiravir, and moxifloxacin in combination or alone. To the best of our knowledge, no study has been conducted to investigate the effects of combined use of these three agents on QTc and QTc dis.

## MATERIALS AND METHODS

### Sample Collection

This study was carried out retrospectively on 193 patients who were hospitalized with the diagnosis of COVID-19 between 11.03.2020 and 01.03.2021. The inclusion criteria of the patient were as follows: having COVID-19 diagnosis by the RNA test through PCR and/or by the computed tomography imaging, being over 18-years-old, and having at least two electrocardiographies which were requested during hospitalization and between the 3^rd^ and 5^th^ days after the initiation of medical treatment due to COVID pneumonia. Patients who were using medications that could affect QRS, QT, and QTc dispersion, tricyclic antidepressants, antipsychotics, antihistamines, patients with previously known branch or atrioventricular nodal block, and those with implantable cardioverter-defibrillators, were excluded from the study. Those with a congenital long QT syndrome and heart rate lower than 60 beats/min or higher than 110 beats/min were also excluded. Patients’ demographic information and laboratory parameters were obtained from their medical records at the hospital.

We compared data of the patients who received favipiravir and HCQ for the treatment of COVID-19 and those who were given moxifloxacin considering bacterial coinfection. We divided the patients into four separate groups due to their medications as, group-1: favipiravir, group-2: favipiravir + HCQ, group-3: favipiravir + moxifloxacin, and group-4: favipiravir + moxifloxacin + HCQ.

The 12-lead ECG recordings were obtained with a 25 mm/s paper speed and 10 mm/mV calibration voltage while the patient in the supine position. Three different cardiologists blind to the clinical data interpreted electrocardiographic parameters. The QT interval was measured from the beginning of the QRS complex to the end of the downslope of the T wave (crossing the isoelectric line); when U wave present, QT interval was measured to the nadir of the curve between the T and U waves. The QT interval corrected for the previous cardiac cycle length (QTc) was calculated according to Bazett’s Formula (QTc=QT/[RR]^1/2^). QTc intervals of 440–460 ms in men and 440–470 ms in women are considered borderline. QTc exceeding the 500 ms threshold or >60 ms prolongation in QTc and QTc dispersion >80 ms compared to baseline ECG was considered to be associated with increased risk of malignant ventricular arrhythmias [6, 7].

This study was conducted in compliance with the ethical standards of the Helsinki Declaration of Human Rights. It was also approved by Turkish Ministry of Health and local ethical committee approval was obtained from the ethical committee of the Umraniye Education and Research Hospital (B.10.1TKH.4.34.H.GP.0.01/117).

### Statistical Analysis

Analyses were performed using the statistical package for the social sciences (SPSS) version 22.0 (SPSS for Windows Inc., Chicago, Illinois, USA). The results of all parameters belonging to patients were given as mean and standard deviation. In the assessment of the study data, the compliance of the parameters with the normal distribution was assessed by using the Kolmogorov–Smirnov test. In addition to descriptive statistics, while considering the study data, the one-way ANOVA test was used to compare the normally distributed parameters among the groups with quantitative data. The Kruskal–Wallis test was used to compare inter-group parameters, not showing normal distribution. Paired t-test was used to compare parameters normally distributed and Wilcoxon test was used to compare parameters between the two related groups. A Chi-square test was used to compare the qualitative data. Probability values were two-tailed and a P-value below 0.05 was considered meaningful.

## RESULTS

In total, 193 patients with the diagnosis of COVID-19 were recruited to this study. The mean age of the patients was 63.1±17.7. Among these patients, 110 (57%) of them were male, and 83 (43%) of the participants were female. The participants were divided into four groups: those using favipiravir (Group 1, n: 54), favipiravir plus HCQ (Group 2, n: 57), favipiravir plus moxifloxacin (Group 3, n: 39), and favipiravir, HCQ, and moxifloxacin (Group 4, n: 43). Hypertension was the most common chronic disease with a 55% incidence of the patients participating in the study. This was followed by coronary artery diseases and diabetes with an incidence of 25%. There was no statistical difference between the groups in terms of baseline characteristics and clinical findings of the patients (Table 1).

**Table 1:** Baseline characteristics and clinical findings of patients

Characteristic	Total (n=193)	Group 1 (n=54)	Group 2 (n=57)	Group 3 (n=39)	Group 4 (n=43)	p
Age, years	63.1±17	62.6±19	52.9±15	68.1±13	65.1±15	0.068
Gender (male/female)	110 /83	28 /26	40 /17	23 /16	19 /24	0.056
Diabetes mellitus, (%)	25	33	17	41	25	0.067
Hypertension, (%)	55	62	56	74	60	0.333
COPD/asthma, (%)	10	9	7	12	16	0.495
CHD (%)	25	33	19	25	25	0.415
Malignancy (%)	5	7	5	7	2	0.679

Group 1: Faviripavir; Group 2: Favipiravir+Hydroxychloroquine; Group 3: Faviripavir+Moxifloxacin; Group 4: Faviripavir+Moxifloxacin+Hydroxychloroquine; COPD: Chronic obstructive pulmonary disease; CHD: Coronary heart disease, Chi-square test.

**Figure 1:**
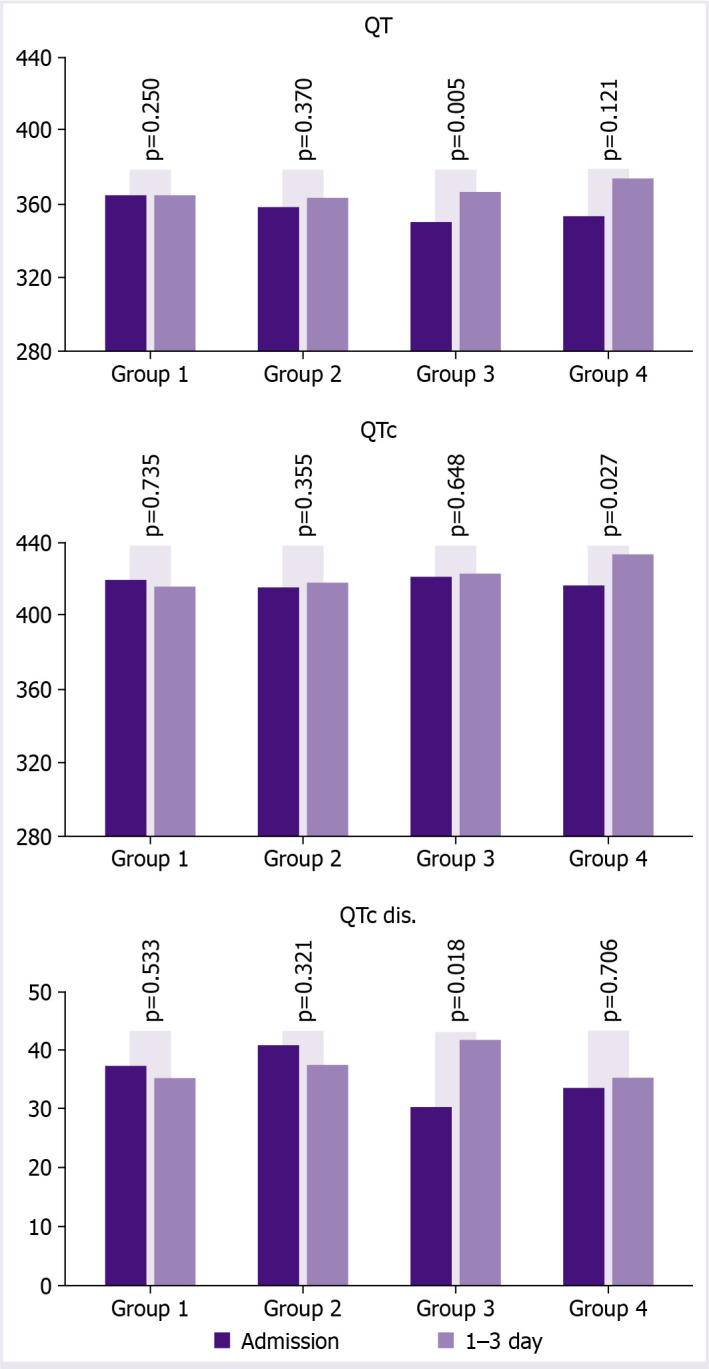
ECG parameter of groups before and after treatment.

The ECGs of the patients which were taken at the time of admission and between the 1^st^ and 3^rd^ days after the initiation of the treatment were evaluated. When the ECGs withdrawn at the time of arrival were evaluated, there was no significant difference between the groups in terms of QT, QTc, and QTc dispersions (p>0.05) (Table 2). When the ECGs taken at the time of arrival and the ECGs taken between the 1^st^ and 3^rd^ day of the start of treatment were evaluated for each group: in group 1, there was a slight increase in the QT value with a decrease in the QTc and QTc dispersion. However, these findings were statistically insignificant (p>0.05). In group 2, although there was a slight decrease in QTc dispersion with an increase in QT and QTc distances, these changes were not statistically significant (p>0.05). In group 3, significant increases in QT and QTc dispersion occurred after using moxifloxacin and favipiravir together (QT [ms]: 350.0±34.6 vs. 366.1±29.0, p=0.005 and QTc dis. [ms]: 30.2±25.3 vs. 41.6±25.4, p=0.018). In the 4^th^ group where the triple therapy was applied, there was no difference in QT and QTc dispersion. On the other hand, there was a significant increase in the QTc values (416.7±34.4 vs. 433.8±44.5; p=0.027) (Fig. 1).

**Table 2:** Comparison of electrocardiographic parameters before and after admission

Parameter	Time of ECG	Group 1 (n=54)	Group 2 (n=57)	Group 3 (n=39)	Group 4 (n=43)	p
QT (ms)	Admission	364.8±44	357.9±29	350.1±34	353.9±37	0.302
	1–3 day	372.2±51	363.4±36	366.2±29	373.6±87	0.822
	P value	0.250	0.370	0.005	0.121	
QTc (ms)	Admission	419.1±42.18	415.5±40.5	421.1±26.9	416.7±34.4	0.894
	1–3 day	416.4±52.1	418.5±47.5	423.6±35.2	433.8±44.5	0.269
	P value	0.735	0.355	0.648	0.027	
QTc dis. (ms)	Admission	37.3±22.6	40.7±23.7	30.2±25.3	33.6±30.3	0.215
	1–3 day	35.2±28.1	37.4±25.8	41.6±25.4	35.2±30.6	0.680
	P value	0.553	0.321	0.018	0.706	

Group 1: Faviripavir; Group 2: Favipiravir+Hydroxychloroquine; Group 3: Faviripavir+Moxifloxacin; Group 4: Faviripavir+Moxifloxacin+Hydroxychloroquine. One-way ANOVA test or Kruskal-Wallis test for inter-group parameters, Paired t-test, Wilcoxon test for the two related groups.

**Figure 2:**
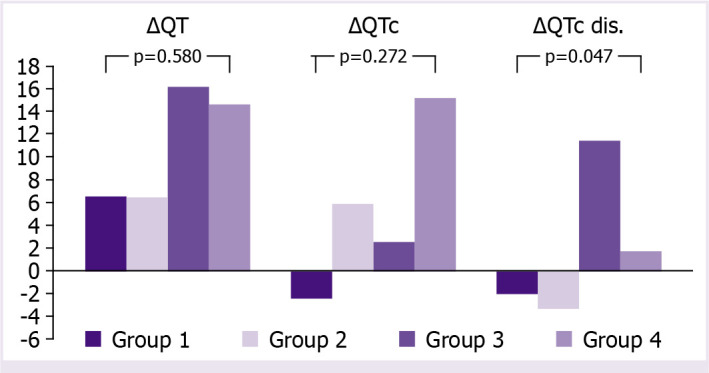
Comparison of electrocardiographic changes for different treatment groups.

When we compared the changes of QT, QTc, and QTc dispersion for each group, although ΔQTc values of the fourth group seem to be higher than the other groups, there was no statistically significant difference for either ΔQT (p=0.580) or ΔQTc (p=0.272) (Table 3). On the other hand, there was statistically significant difference between the groups for ΔQTc dispersion (P: 0.047). *Post hoc* analysis clearly reveals that the reason for this difference is due to the increase in QTc dispersion with the use of moxifloxacin (Group 3; ΔQTc dis.: 11.4±28.8, p=0.047) (Fig. 2).

**Table 3:** Comparison of electrocardiographic changes for different treatment groups

Parameter	Group 1	Group 2	Group 3	Group 4	p
ΔQT	6.4±38.2	6.3±42.1	16.0±32.6	14.5±45.6	0.580
ΔQTc	-2.4±48.1	5.8±45.5	2.5±34.2	15.1±44.4	0.272
ΔQTc dis.	-2.0±25.2	-3.3±24.9	11.4±28.8	1.6±29.0	0.047

Group 1: Faviripavir; Group 2: Favipiravir+Hydroxychloroquine; Group 3: Faviripavir+ Moxifloxacin; Group 4: Faviripavir+Moxifloxacin+Hydroxychloroquine. One-way ANOVA test or Kruskal-Wallis test.

When the QTc exceeding the 500 ms threshold or >60 ms prolongation in QTc and QTc dispersion >80 ms compared to baseline ECG were grouped as risky for malignant ventricular arrhythmias, there were 34 patients considered risky in terms of arrhythmia. When the distribution of patients between the groups were evaluated, there were not significant differences between the groups (group 1: 9 patients [16.7%], group 2: 11 patients [19.3%], group 3: 5 patients [12.8%], group 4: 9 patients [20.9%]; p=0.780).

## DISCUSSION

This study demonstrated that QT, QTc, and QTc dispersion values which were the indicator of arrhythmias might increase differently depending on given medications for COVID-19 patients. Although changes were observed in hydrochloroquine and, to a lesser extent favipiravir, the main agent causing QT changes seems to be the moxifloxacin. QTd as a measure of myocardial inhomogeneity was also significantly higher in the group using moxifloxacin rather than the other groups.

Favipiravir is an antiviral derived from pyrazine carboxamide that was initially used to treat influenza in Japan [8]. A unique characteristic of favipiravir is its broad-spectrum activity toward RNA viruses, including influenza virus, rhinovirus, respiratory syncytial virus, and Ebola virus [9, 10]. Nowadays, due to its inhibition of RNA polymerase, favipiravir thought to have potential antiviral activity on SARS-CoV-2 infection [11]. In various studies, favipiravir has been suggested to have greater antiviral activity than lopinavir/ritonavir and approved for SARS-CoV-2 treatment since February 15, 2020 [12]. As with other medications used in the treatment of COVID-19, several publications have been published on the antiarrhythmic effects of favipiravir, with contradictory conclusions. In a case report, an increase in the QT (320 ms vs 480 ms) and QTc (378 ms vs. 476 ms) levels was observed after using 1, 2 grams of favipiravir twice a day after 6 grams of loading dose for Ebola virus treatment [13]. Although this patient had used drugs that could cause QT prolongation such as levofloxacin, it was stated that ECG changes could be due to the use of high-dose favipiravir since levofloxacin was discontinued before treatment of favipiravir. Despite all these data, it was added by the author that QT changes might also be caused by the effect of levofloxacin at the tissue level [13]. In another study, Kumagai et al. [5] had administered a single dose of 1200 mg, and 2400 mg favipiravir, and 400 mg moxifloxacin to healthy adult Japanese in different groups to compare their ECG changes; they had found no effect of favipiravir on QT intervals. Çap et al. [14] had demonstrated that the effect of favipiravir use (1600 mg BID for 1^st^-day loading dose followed by 600 mg BID for 4 days) did not have a significant effect on QT parameters. In our study, as in the study of Çap et al. [14], we could not found any change in QT and QTc parameters in patients using just only favipiravir. We also evaluated the QTc dispersions, and although it was not statistically significant, a slight decrease was observed with the initiation of favipiravir treatment (−2.0±25.2 ms).

Chloroquine and HCQ have been commonly used in the treatment and prophylaxis of malaria and chronic rheumatic diseases since 1934. Although chloroquine and HCQ have been reported to be effective against viruses, including SARS-CoV-2, on the contrary, some other studies have indicated that HCQ is not effective in the treatment of COVID-19, as it did not reduce intubation or death rate [15–19]. Since the effectiveness of the treatment is open to debate, although they are frequently used in the early stages of the disease, recently, they are not included in the treatment protocols. In addition to studies about their effectiveness, various studies show that chloroquine and HCQ are proarrhythmic agents and cause significant QT prolongation [20, 21]. HCQ exerts its cardiac effect by inhibiting sodium, potassium, and calcium channels (IK1, IKr, and hERG). Inhibition of Na and Ca current results widening of the QRS, whereas the inhibition of K influx results in QTc prolongation [22].

In most of the studies, although significant prolongations in QT interval were observed with Chloroquine, relatively less prolongations in QT interval were observed with HCQ, which is a less toxic metabolite of chloroquine [23]. Studies have shown prolongations in the QT interval between 5 and 6 ms, generally 1–3 days after the initiation of treatment [14, 15, 24]. On the other hand, in the study of Sridhar et al. [25], the ΔQTc distance has been decreased as −25±24 ms. Although many studies can be found related with QT prolongation in the literature, QTd, which is an indicator of ventricular repolarization, has not been studied so much. In the study of Isik et al. [26], the median QTc dispersion increased from 44 to 46 ms after HCQ administration [26]. Meanwhile, in the same study, 12 of 77 (15%) patients showed a prolongation of QTc dispersion >60 ms, which is important in terms of the risk of developing ventricular arrhythmia. In our study, in accordance with most studies, increases in QT and QTc distances (ΔQT: 6.3±42.1 and ΔQTc: 5.8±45.5) were observed, but they were not found to be statistically significant. When QTc dispersion was evaluated, unlike the study of Isik et al. [26], although it was not statistically significant, our findings showed a decrease in QTc dispersion rather than an increase (QTC disp.; 40.7±23.7 ms at admission vs. 37.4±25.8 ms after HCQ. p=0.321).

Moxifloxacin is a fluoroquinolone group antibiotic that exerts its effects by targeting the bacterial DNA gyrase and topoisomerase IV, thus inhibiting bacterial synthesis of DNA and leading to rapid bacterial death [27]. Fluoroquinolones are active against gram-negative and gram-positive bacteria, anaerobes, mycobacteria, and atypical pathogens. Respiratory fluoroquinolones, levofloxacin, and moxifloxacin constitute fist-line therapeutic agents for the management of severe community-acquired pneumonia [28]. They are chemical derivatives of quinoline, and quinolone-based compounds have been investigated for their antiviral activity against various viruses such as Ebola and Dengue virus, papovavirus, human cytomegalovirus, varicella-zoster virus, herpes simplex virus types 1 and 2, hepatitis C virus, and HIV [28–32]. A recent *in silico* study demonstrated that the fluoroquinolones, ciprofloxacin, and moxifloxacin may inhibit SARS-CoV-2 replication [33].

Besides its antimicrobial activity, moxifloxacins have similar effects as HCQ by dose-dependent blocking of the rapid activating delayed rectifier potassium current (IKr) encoded by the human ether-a-go-go-related gene (HERG, thereby causing the prolongation of the QT interval and TdP) [34].

Based on previous meta-analyses and publications, QT prolongation or Torsaes de pointes can be observed in approximately 5.5 and 7% of moxifloxacin-induced cardiovascular side effects [35]. Frothingham et al. [36] reported that fluoroquinolones cause QT prolongation and TdP; the highest risk appears to be with gatifloxacin followed by levofloxacin, ofloxacin, and ciprofloxacin and moxifloxacin. These studies associated with moxifloxacin indicated that the QT interval prolongation may range from 6 ms to 20 ms [35–40]. Although case reports of moxifloxacin-induced QT prolongation and subsequent TdP generally resolved spontaneously, defibrillation was needed in some cases [37, 41]. According to the results of previous studies, the risk of QT prolongation may be higher with the use of moxifloxacin in patients with multiple risk factors such as advanced age, female gender, administration of one or more QT-prolonging drug, and comorbid risk factors.

In our study, in the third group, patients who were used moxifloxacin together with faviripavir evaluated. To the best of our knowledge, there is no other study in the literature that evaluated changes in QT intervals after the use of faviripavir and moxifloxacin. As we mentioned before, since favipiravir did not cause significant changes on QT parameters in the group in which it was used alone, it can be considered that the changes that occur after its use with moxifloxacin were generally caused by moxifloxacin. In our study, after the use of moxifloxacin and favipiravir together, significant prolongation of the QT interval (350.0±34.6 vs 366.1±29.0 p=0.005) occurred, which is consistent with previous studies mentioning the moxifloxacin and its cardiac effects. In addition, although there was no statistically significant change in QTc values, a significant increase in Qtc dispersion was observed (QTc dis.: 30.2±25.3 vs. 41.6±25.4, p=0.018). When the changes of QT parameters compared (Table 3) among the groups, there was a difference only in the ΔQTc dispersion parameter, which seems to be due to the differences of the third group (ΔQTc dis.: 11.4±28.8 for group 3, p=0.047).

Since COVID-19 first appeared, many combined therapies have been tried successfully or unsuccessfully. The combination of HCQ and azithromycin has often been used to increase the effectiveness of HCQ, but many case reports have been demonstrated the prolongation of the QT interval and TdP [14, 15, 42]. Other than azithromycin, although there are publications regarding the antiviral activity of moxifloxacin, it has been used together with HCQ if it is thought that a bacterial infection accompanies the viral infection in order to benefit from its antibacterial activity in routine practice. In the study of Afsin et al. [4], a significant increases in QT and QTc were observed following concomitant use of moxifloxacin and HCQ (370.0+/32.5 − 381+/−29 p<0.001). Despite the prolongation of the QT interval, Afsin et al. [4] stated that the combined use of HCQ and moxifloxacin is safe. In our study, a significant increase in QTc distance was observed with the combined use of faviripavir, moxifloxacin, and HCQ (416.7±34.4 vs. 433.8±44.5 p=0.027). Although the changes in ΔQTc distance were not statistically significant between the groups, they were obviously higher in this group (Group 4 ΔQTc: 15.1±44.4) (Fig. 2).

When we grouped the patients being high risk for arrhythmia as they had QTc exceeding the 500 ms threshold or >60 ms prolongation in QTc and QTc dispersion >80 ms, 34 over 193 patients considered risky in terms of arrhythmia without any significant differences between the four groups (p=0.780). The lack of significant difference between the groups suggests that the condition of the patients might be due to the cardiac effects of the disease itself rather than the medications they used.

The limitations of our study are that it was retrospective and was conducted with a small number of patient groups. This study might be planned prospectively with a large number of sample groups.

## Conclusion

In our study, although different QT interval changes were seen due to different treatment regimens, we could not see any case of significant ventricular arrhythmia or TdP. Although HCQ has some effects on QT parameters, moxifloxacin seems to be the main drug causing QT changes. Besides the use of HCQ decreased according to the latest guidelines, if there is a COVID-19 infection with an additional bacterial infection, and if there is a need of using HCQ together with moxifloxacin, additional risk factors that may cause QT prolongation should be reviewed and ECG monitoring of the patients should be performed during treatment period.
